# Sarcopenia in liver transplantation: A comprehensive bibliometric study of current research trends and future directions

**DOI:** 10.1515/biol-2025-1197

**Published:** 2025-10-27

**Authors:** Yang Li, Yang Xiang, Xiaoyan Yan, Changjiang Lu, Jing Huang, Lei Dai

**Affiliations:** Department of Emergency, Ningbo Medical Centre Lihuili Hospital, The Affiliated Hospital of Ningbo University, Ningbo, Zhejiang, 315040, China; Department of Hepato-Pancreato-Biliary Surgery, Ningbo Medical Centre Lihuili Hospital, The Affiliated Hospital of Ningbo University, 1111 Jiangnan Road, Ningbo, Zhejiang, 315040, China; Health Science Center, Ningbo University, Ningbo, 315211, China; Outpatient Department, Ningbo Medical Centre Lihuili Hospital, The Affiliated Hospital of Ningbo University, Ningbo, Zhejiang, 315040, China

**Keywords:** sarcopenia, liver transplantation, bibliometric analysis, nutrition, targeted therapy

## Abstract

The long-term survival and quality of life of liver transplantation (LT) recipients has emerged as a critical focus, where managing sarcopenia (a syndrome of diminished muscle mass and strength) and perioperative nutrition is paramount. This study aimed to delineate the knowledge landscape and identify key research trends and potential molecular mechanisms linking LT and sarcopenia through bibliometric and bioinformatics analyses. This study employs bibliometric and bioinformatics analyses to evaluate research trends and molecular mechanisms linking LT and sarcopenia. Data were retrieved from Web of Science database, and tools such as CiteSpace, VOSviewer, and R were used for data analysis and visualization. A total of 448 studies published over the past two decades were analyzed. Our bibliometric analysis revealed geographical distribution patterns, authorship networks, journal contributions, and thematic trends related to both LT and sarcopenia. Bioinformatics analysis identified 78 biphenotypic target genes shared by LT and sarcopenia, with hub genes including IL1B, ADIPOQ, and TNF showing strong associations. Enrichment analyses further highlighted significant biological processes, such as “response to peptide hormone” and “regulation of glucose transmembrane transport,” suggesting potential molecular mechanisms underlying the interaction between LT and sarcopenia. This study highlights the importance of incorporating routine sarcopenia assessments into the clinical management of LT candidates to optimize treatment strategies. Future research should focus on elucidating the molecular pathways connecting these conditions and developing targeted interventions to improve LT patients’ outcomes and quality of life.

## Graphical abstract

**Figure:**
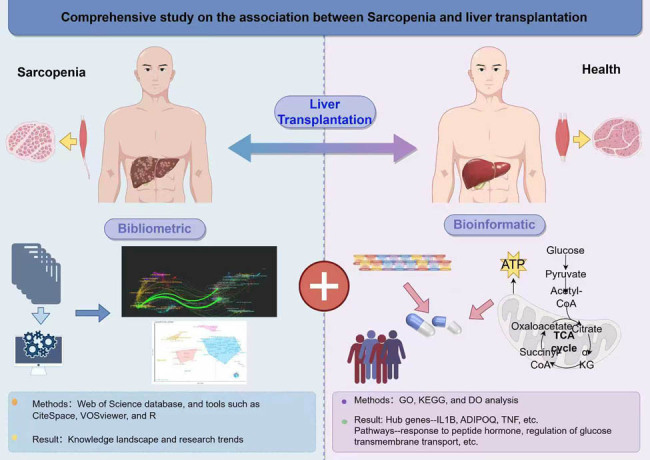

## 1 Introduction

Liver transplantation (LT) is an important therapeutic option for individuals with end-stage liver disease [1–3], considerably improving survival rates and quality of life [4]. Nonetheless, post-transplant complications are common among recipients [5], with muscle wasting diseases such as sarcopenia being particularly prevalent [6]. Sarcopenia, characterized by gradual loss of skeletal muscle mass and strength [7,8], has been identified as a negative factor that can impede recovery and shorten the overall survival in LT patients [9]. The relationship between LT and sarcopenia is complex, underscoring the necessity for further research to elucidate their interplay and potentially improve patient outcomes and inform clinical approaches. Recent studies in this domain have highlighted the importance of investigating molecular mechanisms underlying sarcopenia development in the context of LT [10].

Recent studies have demonstrated that preoperative muscle mass serves as a significant predictor of postoperative outcomes [11,12]. Sarcopenic patients exhibit higher rates of complications and mortality compared to their non-sarcopenic counterparts [13,14]. This correlation emphasizes the importance of personalized therapies aimed at preserving muscle mass before and after LT. However, the present research shows substantial gaps in our understanding of biochemical pathways and genetic variables related to sarcopenia in LT patients.

To overcome these challenges, we propose a multimodal research framework that integrates bibliometric analysis with biological mechanism investigation. Bibliometric analysis sheds light on the trends and hotspots in the research landscape around LT and sarcopenia [28–30], elucidating key patterns in publication output, authorship, and institutional contributions. This quantitative assessment establishes a foundation for identifying major study themes and prospective areas for future exploration. Concurrently, biological mechanism analysis can uncover the underlying genetic and molecular networks linking sarcopenia to LT, thereby revealing promising treatment targets.

The purpose of this work is to comprehensively analyze the association between LT and sarcopenia using a dual methodological approach that combines bibliometric insights with biological interventions. By reviewing the available literature, we aim to identify key genes and molecular pathways associated with both disorders, potentially uncovering biomarkers for early detection and intervention. Furthermore, this study seeks to enhance the current understanding of sarcopenia’s impact on liver transplant outcomes, ultimately guiding clinical practice toward more effective management strategies for at-risk populations.

To our knowledge, existing studies on sarcopenia in LT patients remain predominantly narrative, focusing on clinical features and management. While informative, they offer limited quantitative insight into the evolution of the field or the molecular mechanisms linking LT and sarcopenia. Our study introduces an integrative approach to address these gaps: (1) we employ bibliometrics to objectively map 20 years of research trends, collaborations, and knowledge foundations, providing a macro-analytic perspective beyond traditional reviews; (2) we further analyze transcriptomic data to identify novel hub genes and signaling pathways that may mechanistically connect LT to sarcopenia, offering new directions for basic and translational research. This dual methodology combines science mapping with mechanistic discovery, advancing the current literature both quantitatively and biologically.

## 2 Materials and methods

### 2.1 Data processing

**Figure 1:**
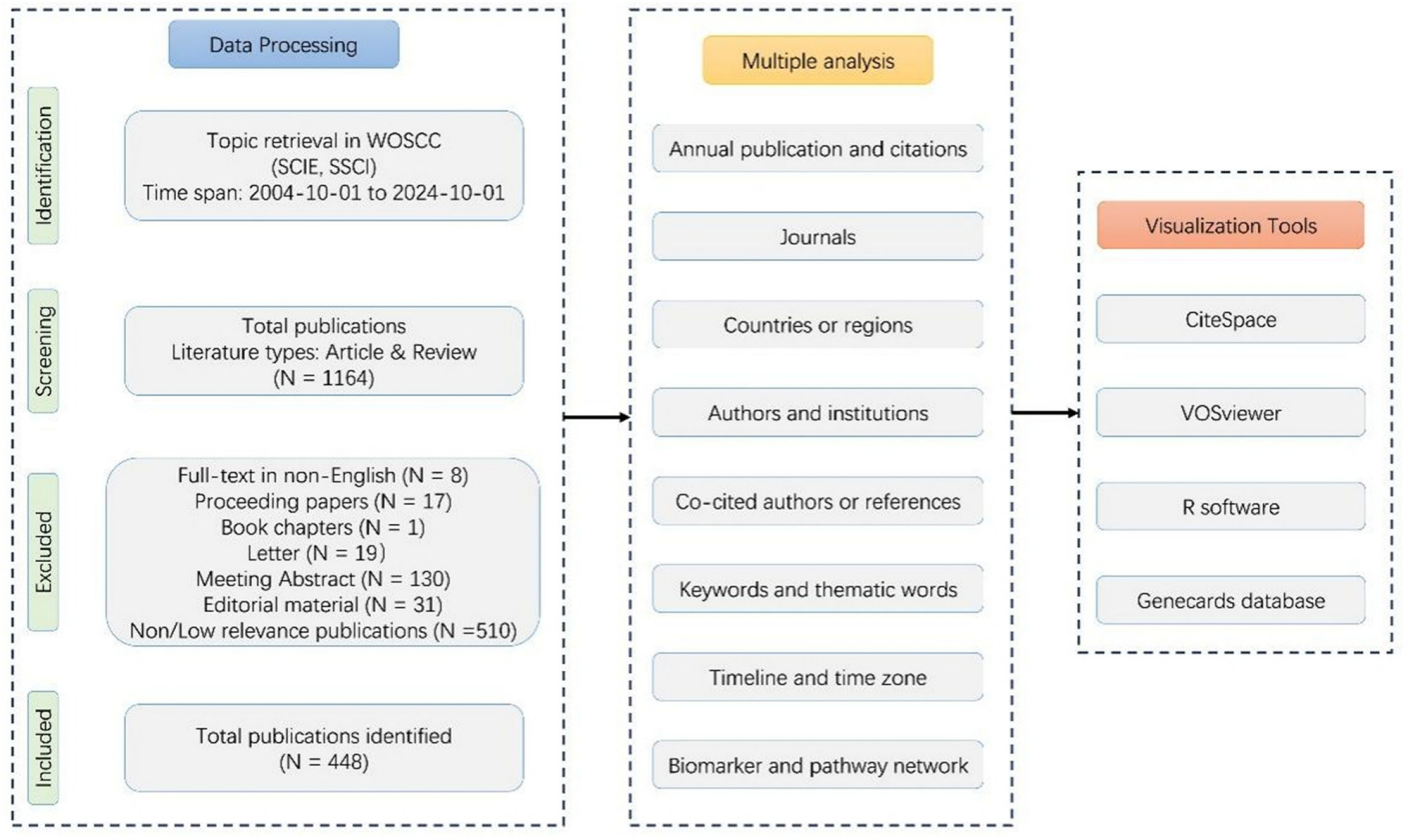
Flowchart of bibliometric analysis. The flowchart demonstrated the detailed search strategy, inclusion and exclusion criteria, and analysis software. WOSCC: web of science core collection; SCIE: science citation index expanded; SSCI: social science citation index.

The Web of Science (Clarivate Analytics, Philadelphia, USA) (https://webofscience.clarivate.cn/wos/alldb/basic-search) is a premier database for bibliometric analyses, recognized for its broad disciplinary coverage, precise citation indexing, and diverse analytical metrics [15,16]. These features enable researchers to identify emerging themes and trends in their respective fields. For our bibliometric analysis, we extracted publication data on LT and sarcopenia from the Web of Science Core Collection (WoSCC), specifically utilizing the Science Citation Index Expanded (SCIE) and Social Sciences Citation Index (SSCI) editions. To minimize bias arising from data updates, all search, extraction, and downloading activities were conducted on the same day. Our literature review focused exclusively on articles and reviews, with the search methods, results, and terminology [17–19] detailed in Figure 1.

### 2.2 Search strategy

The query formulation was as follows: (1) TS = (sarcopen* OR myopeni* OR dynaponi*); (2) TS = (muscle OR muscular); (3) TS = (atroph* OR weak* OR depletion* OR loss* OR wasting*); (4) 2 AND 3; (5) 1 OR 4; (6) TS = (LT) OR (liver transplant*) OR (Liver Grafting) OR (Grafting, Liver) OR (Liver Transplant*) OR (Transplantation, Liver) OR (Transplant, Liver) OR (Transplantation, Hepatic) OR (Hepatic Transplantation*) OR (hepatic graft∗); (7) 5 AND 6. Then, three researchers (one is a Chief Physician from the LT department, one is a PhD in hepatology, and one is a senior clinical nutrition assessment specialist, all with over 10 years of experience in the field and substantial expertise in systematic literature review) independently evaluated each article to ensure the accuracy and consistency of the pre-processed data [20]. In total, 448 relevant publications were identified, and the original data, which included full records and cited references, were retrieved in text format.

### 2.3 Multiple analyses and visualization

In this study, we conducted a comprehensive multi-dimensional analysis of research landscape in the field of sarcopenia and LT, including publication quantity and growth rate, leading countries and institutions, high-contributing authors and journals, frequently-cited references, temporal trends of active authors and publications, keyword bursts and clustering, and thematic trends. The analyses and visualizations were conducted using Excel (version 2016, Microsoft, DC, USA) [21], R software (version 4.2.1) (https://www.r-project.org/) with the bibliometrix package [22], CiteSpace (version 6.3.R1, 64-bit, advanced) (https://citespace.podia.com/) (java-based high-performance scientific literature analysis tool) [23], and visualization of similarities viewer (VOSviewer) (version 1.6.20, CWTS, Leiden University, The Netherlands) (https://www.vosviewer.com/) (a free, open-source software for bibliometrics and scientific visualization) [24]. This multi-dimensional approach effectively elucidates the current status, focus, and trends of sarcopenia in the context of LT, providing valuable insights to enhance the understanding of this field’s dynamics and provide information on future research.

### 2.4 Biological mechanism analysis

GeneCards, The Human Gene Database (Weizmann institute of Science, Rehovot, Israel) (https://www.genecards.org/) [25], is a user-friendly knowledge base that provides comprehensive information on human multiple-omics data. Using this tool, we successfully retrieved gene sets associated with sarcopenia and LT. For each gene set, we filtered genes categorized as “protein coding” and a relevance score greater than 1 and performed an intersection analysis to identify genes common to both LT and sarcopenia. Subsequently, the Friends analysis [26] was employed to calculate the importance of each gene based on network topology parameters and further analyze their functions and regulatory mechanisms in relevant biological processes (BPs). Hub genes were identified from the target genes using this analysis and visualized via a cloud plot.

Furthermore, we performed enrichment analysis of hub genes, including Gene Ontology (GO), Kyoto Encyclopedia of Genes and Genomes (KEGG) [27], and Disease Ontology (DO) enrichment analyses [28]. Using the “DOSE” and “org.Hs.eg.db” packages (version 3.24.2), we explored potential disease categories related to the hub genes in DO analysis. GO analysis provided detailed insights into the BPs, cellular components (CCs), and molecular functions (MFs) associated with the hub genes, while KEGG enrichment analysis investigated the pathways and mechanisms linked to these genes.

Additionally, we analyzed the functional protein association networks of the hub genes using the STRING database (https://cn.string-db.org/) [29]. The analysis was performed with the following parameters: maximum false discovery rate ≤0.05, minimum signal ≥0.01, and minimum strength ≥0.01. Reactome pathway analysis [30] and tissue expression (TISSUES) enrichment analyses were also conducted. These analyses collectively suggested potential pathways and future research directions from a biological mechanism perspective.

## 3 Results

Figure 1 illustrates the detailed workflow and results of the literature identification and screening process.

### 3.1 Worldwide overview

According to our search strategy, a total of 448 studies related to LT and sarcopenia (321 articles and 127 reviews) were retrieved from the WoSCC database over the past two decades. These publications received significant contributions from 47 countries and regions, 664 institutions, 2,546 authors, and 146 journals.

### 3.2 Publication metrics analysis

**Figure 2:**
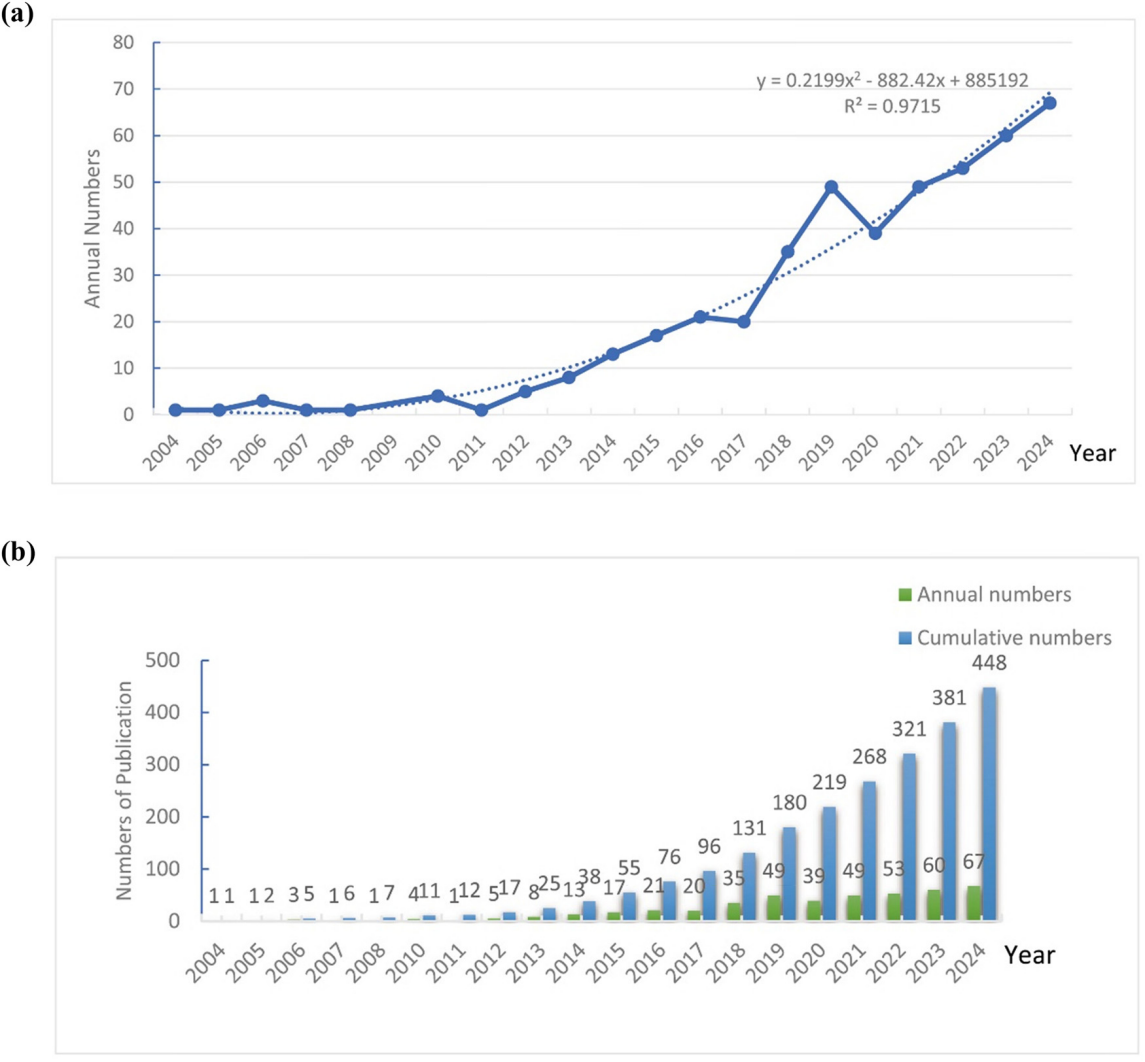
Distribution and growth trend of publications about sarcopenia and LT. (a) Increasing trends in annual publications from 2004 to 2024 with a polynomial function and fitted curve by using Microsoft Excel 2019. (b) Annual cumulative number of publications from 2004 to 2024.

By evaluating the number of publications in the fields of LT and sarcopenia over the years, we visualized the publication trends using Excel (version 2019) and constructed a bivariate linear equation to predict future trends (Figure 2a):

\[y=0.2199{t}^{2}-882.42t+885192\text{}({R}^{2}=0.9715).]\]

Based on this model, the field is expected to experience sustained exponential growth in the coming years. Additionally, we identified 2014 as a significant turning point, dividing the publication growth rate and total volume into two phases: Period I (2004–2013) and Period II (2014–2024). During Period I, the annual publication volume was below ten, with the growth rate approaching zero. However, since 2014, publications have grown rapidly, with the volume in 2024 expected to be 3.15 times higher than that in 2014. Furthermore, the total volume over the next decade is predicted to increase by a factor of 9.79 compared to the previous decade (Figure 2b). This accelerated growth suggests that the field is garnering increasing scientific attention, which is likely to accelerate the overcoming of existing research bottlenecks.

### 3.3 Analysis of the distribution of countries and institutions

**Figure 3:**
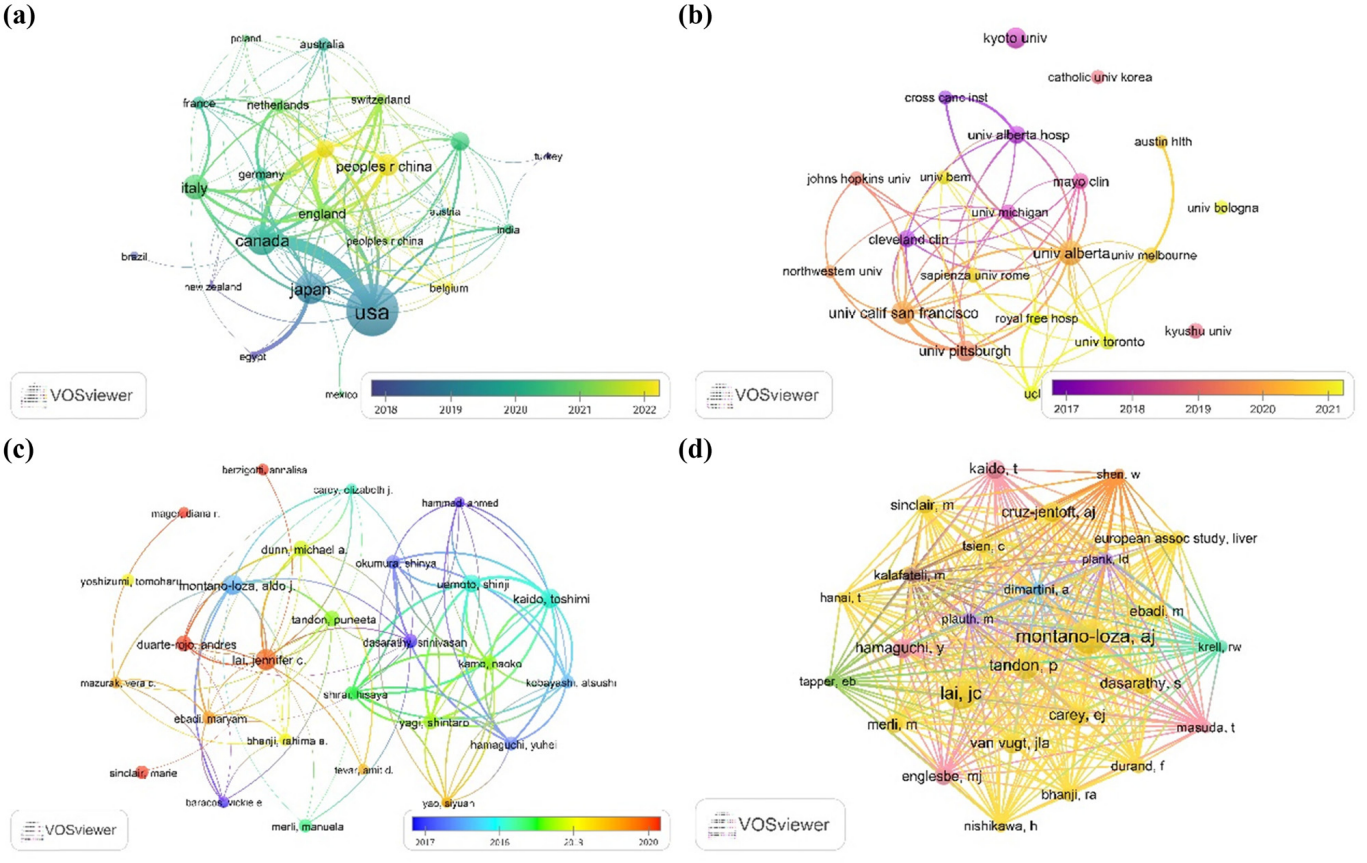
Network visualization of geography, institutions, and authors on research related to sarcopenia and LT. (a) Network map of countries on research of sarcopenia and LT. (b) Network map of institutions on research of sarcopenia and LT. (c) Network map of authors on research of sarcopenia and LT. (d) Network map of co-cited authors on research of sarcopenia and LT.

Our analysis revealed that the total number of publications from the top ten countries in this field was nearly three times greater than the total output from other countries. Notably, the United States led in both publication volume (*n* = 130) and citation count (*n* = 7,354). Among the top ten countries, 70% were from Europe and North America, while 30% were from Asia, with the publications of the former exceeding those of the latter by a factor of 2.4. Among Asian countries, Japan had the highest publication volume (*n* = 61) and citation count (*n* = 2,451), while China ranked fifth with 43 publications and 693 citations but had the lowest average citation per publication (16.12). Although Canada ranked third in publication volume, it held the highest average citation per publication (70.85) (Table 1). Additionally, overlay visualization revealed that publications from the United States and Japan were relatively early (2018–2019), while other European and North American countries published mainly during 2020–2021. China saw a sharp increase in publication volume starting in 2022 (Figure 3a). Network analysis also showed that international collaborations were primarily concentrated among the top ten countries.

**Table 1:** Top ten countries and institutions on research of sarcopenia and LT

Rank	Country	Documents	Citations	Average number of citations/publications	Institution	Documents	Citations	Average number of citations/publications
1	USA	130	7,354	56.57	University of Alberta	34	2,273	66.85
2	Japan	61	2,451	40.18	University of California, San Francisco	30	1,941	64.70
3	Canada	59	4,180	70.85	University of Pittsburgh	24	1433	59.71
4	Italy	43	1,335	31.05	Kyoto University	23	1,385	60.22
5	Peoples’ R China	43	693	16.12	University of Alberta Hospital	15	2,034	135.60
6	South Korea	29	682	23.52	Cleveland clinic	13	1,737	133.62
7	Spain	27	514	19.04	University of Toronto	12	336	28.00
8	England	26	715	27.50	Mayo clinic	12	854	71.17
9	The Netherlands	17	837	49.24	UCLA	11	494	44.91
10	Germany	17	671	39.47	University of Michigan	11	1,221	111.00

Half of the top ten contributing institutions in this field were based in the United States, with only one institution from Japan, Kyoto University (Rank 4, 23 publications and 1,385 citations). The University of Alberta in Canada has produced the highest number of publications in the last 20 years (34 publications and 2,273 citations). However, in terms of average citation per publication, University Alberta Hospital (135.60), Cleveland Clinic (133.62), and University of Michigan (111.00) all exceed 100, far outperforming other institutions (Table 2). Similarly, we found that these leading institutions published relatively early, mainly between 2017 and 2019. Moreover, network analysis revealed that institutions in North America had close collaborative ties, while Japanese institutions were relatively isolated (Figure 3b).

**Table 2:** Top ten authors and co-cited authors on research of sarcopenia and LT

Rank	Author	Documents	Citations	Average number of citations/publications	H-index	G-index	Co-cited authors	Citations
1	LAI, JENNIFER C.	27	1,908	70.67	18	27	MONTANO-LOZA, ALDO J.	524
2	MONTANO-LOZA, ALDO J.	24	3,457	144.04	22	25	LAI, JENNIFER C.	442
3	KAIDO, TOSHIMI	22	1,082	49.18	14	23	TANDON, PUNEETA	317
4	UEMOTO, SHINJI	20	1,059	52.95	13	20	CAREY, ELIZABETH J	215
5	YAGI, SHINTARO	15	412	27.47	9	15	DASARATHY, SRINIVASAN	198
6	TANDON, PUNEETA	15	1,482	98.80	13	15	VAN VUGT, JEROEN L. A.	189
7	DUARTE-ROJO, ANDRES	15	911	60.73	11	15	CRUZ-JENTOFT AJ	188
8	DUNN, MICHAEL A.	14	1,049	74.93	13	14	HAMAGUCHI, YUHEI	180
9	KAMO, NAOKO	13	386	29.69	9	13	KAIDO, TOSHIMI	174
10	SHIRAI, HISAYA	12	394	32.83	9	12	SINCLAIR, MARIE	168

### 3.4 Analysis of authors’ and co-cited authors’ distribution

The top ten contributing authors were predominantly from medical institutions in the United States, Canada, and Japan, with half of them being Japanese scholars. An interesting observation was that these five Japanese scholars were all affiliated with Kyoto University and collaborated closely with one another. They had co-authored a total of 82 publications in the fields of LT and sarcopenia, which had been cited 3,333 times. To further assess their academic impact, we used the *H*-index (which balances the quantity and overall quality of an author’s publications) and the *G*-index (which emphasizes the influence of highly cited articles). Among the top contributors, LAI, JENNIFER C from the University of California, San Francisco, USA, had published the most articles (*n* = 27) and had the highest *G*-index (*n* = 27). Meanwhile, MONTANO-LOZA, ALDO J from the University of Alberta in Canada had the most citations (*n* = 3,457), with an average citation per publication of 144.04, an *H*-index of 22, and a *G*-index of 25, all of which were high (Table 2). Additionally, network analysis revealed that Japanese scholars published earlier (2017–2018), while their European and American counterparts focused more on publications post-2019, and there was a lack of close academic collaboration between the two groups (Figure 3c).

We further explored the collaborative relationships within the academic community using co-citation analysis, which refers to the simultaneous citation of two or more articles by subsequent research. Among the top ten authors with high contributions, four were also with high co-citations. MONTANO-LOZA, ALDO J had the highest co-citation count (*n* = 524), followed by LAI, JENNIFER C (*n* = 442) and TANDON, PUNEETA (*n* = 317) (Table 2). Similar to the high-contributing authors, the high co-cited authors were primarily clustered into two categories: one consisting of European and American scholars and the other of Japanese scholars (Figure 3d).

### 3.5 Analysis of references and co-cited references

We summarized the key information of the top ten highly cited and highly co-cited references in Tables 3 and 4. The results showed that all of the top ten highly cited references had been cited more than 250 times, with the first-ranked reference by ENGLESBE MJ (DOI: 10.1016/j.jamcollsurg.2010.03.039) and the second-ranked reference by MONTANO-LOZA AJ (DOI: 10.1016/j.cgh.2011.08.028), both having been cited over 600 times. All references focused on the assessment of muscle mass and prognostic factors in LT patients. Notably, three references proposed integrating the model for end-stage liver disease (MELD) score with sarcopenia into the LT evaluation system (seventh, nineth, and tenth ranks), while two others suggested criteria or thresholds for muscle wasting (fifth and sixth ranks) (Table 3).

**Table 3:** Top ten references with highest citations on research of sarcopenia and LT

Rank	Author	Title	Key point	DOI	Total Citation	Journal	Year
1	ENGLESBE MJ	Sarcopenia and mortality after liver transplantation	Sarcopenia assessment	10.1016/j.jamcollsurg.2010.03.039	621	J AM COLL SURGEONS	2010
			LT outcomes				
			Psoas muscle area				
			Post-transplantation mortality				
			Patient frailty indicators				
2	MONTANO-LOZA AJ	Muscle wasting is associated with mortality in patients with cirrhosis	Sarcopenia prevalence	10.1016/j.cgh.2011.08.028	606	CLIN GASTROENTEROL H	2012
			Liver cirrhosis				
			Mortality prediction				
			LT evaluation				
			Muscle mass assessment				
3	TANDON P	Severe muscle depletion in patients on the liver transplant wait list: its prevalence and independent prognostic value	Sarcopenia prevalence	10.1002/lt.23495	430	LIVER TRANSPLANT	2012
			LT candidates				
			Prognostic significance				
			Waiting list mortality				
			Muscle depletion predictors				
4	DASARATHY S	Sarcopenia from mechanism to diagnosis and treatment in liver disease	Sarcopenia in cirrhosis	10.1016/j.jhep.2016.07.040	396	J HEPATOL	2016
			Muscle mass assessment				
			LT outcomes				
			Mortality risk				
			Therapeutic targets				
5	HAMAGUCHI Y	Proposal for new diagnostic criteria for low skeletal muscle mass based on computed tomography imaging in Asian adults	Sarcopenia assessment in LT	10.1016/j.nut.2016.04.003	361	NUTRITION	2016
			Psoas muscle mass index (PMI) as a predictor				
			Sex-specific cutoff values for low skeletal muscle mass				
			Sarcopenia’s impact on post-transplant survival				
			Asian population criteria for sarcopenia				
6	CAREY EJ	A multicenter study to define sarcopenia in patients with end-stage liver disease	Sarcopenia definition	10.1002/lt.24750	339	LIVER TRANSPLANT	2017
			LT				
			SMI				
			Wait-list mortality				
			Muscle wasting thresholds				
7	LAI JC	Development of a novel frailty index to predict mortality in patients with end-stage liver disease	Frailty index for cirrhosis	10.1002/hep.29219	330	HEPATOLOGY	2017
			LT candidates				
			Mortality prediction				
			MELD score supplementation				
			Extrahepatic complications				
8	KAIDO T	Impact of Sarcopenia on Survival in Patients Undergoing Living Donor Liver Transplantation	Sarcopenia assessment	10.1111/ajt.12221	303	AM J TRANSPLANT	2013
			Living donor liver transplantation (LDLT)				
			Post-transplantation mortality				
			Muscle mass correlation				
			Perioperative nutritional therapy				
9	DURAND F	Prognostic value of muscle atrophy in cirrhosis using psoas muscle thickness on computed tomography	Sarcopenia and mortality	10.1016/j.jhep.2014.02.026	284	J HEPATOL	2014
			LT				
			MELD score				
			Psoas muscle thickness (TPMT)				
			Prognostic marker				
10	VAN VUGT JLA	Systematic Review and Meta-analysis of the Impact of Computed Tomography–Assessed Skeletal Muscle Mass on Outcome in Patients Awaiting or Undergoing Liver Transplantation	Sarcopenia assessment	10.1111/ajt.13732	260	AM J TRANSPLANT	2016
			LT outcomes				
			MELD score supplementation				
			Muscle waste as a prognostic marker				
			Preoperative skeletal muscle mass and survival				

**Table 4:** Top ten co-cited references on research of sarcopenia and liver transplantation

Rank	Author	Title	Key point	DOI	Total Citation	Journal	Year
1	MONTANO-LOZA AJ	Muscle wasting is associated with mortality in patients with cirrhosis	Sarcopenia prevalence	10.1016/J.CGH.2011.08.028	144	CLIN GASTROENTEROL H	2012
			Liver cirrhosis				
			Mortality prediction				
			LT evaluation				
			Muscle mass assessment				
2	ENGLESBE MJ	Sarcopenia and mortality after liver transplantation	Sarcopenia assessment	10.1016/J.JAMCOLLSURG.2010.03.039	138	J AM COLL SURGEONS	2010
			LT outcomes				
			Psoas muscle area				
			Post-transplantation mortality				
			Patient frailty indicators				
3	TANDON P	Severe muscle depletion in patients on the liver transplant wait list: Its prevalence and independent prognostic value	Sarcopenia prevalence	10.1002/LT.23495	119	LIVER TRANSPLANT	2012
			LT candidates				
			Prognostic significance				
			Waiting-list mortality				
			Muscle depletion predictors				
4	CAREY EJ	A multicenter study to define sarcopenia in patients with end-stage liver disease	Sarcopenia definition	10.1002/LT.24750	109	LIVER TRANSPLANT	2017
			LT				
			SMI				
			Wait-list mortality				
			Muscle wasting thresholds				
5	MONTANO-LOZA AJ	Severe muscle depletion predicts postoperative length of stay but is not associated with survival after liver transplantation	Sarcopenia in cirrhosis	10.1002/LT.23863	105	LIVER TRANSPLANT	2014
			LT outcomes				
			Mortality prediction				
			Post-transplant complications				
			Muscle mass assessment				
6	CRUZ-JENTOFT AJ	Sarcopenia: European consensus on definition and diagnosis	Sarcopenia definition and diagnosis	10.1093/AGEING/AFQ034	97	AGE AGEING	2010
			European Working Group on Sarcopenia in Older People (EWGSOP) criteria				
			Muscle mass and function assessment				
			Clinical utility and statistical accuracy balance				
			Mortality prediction in cirrhosis patients				
7	DURAND F	Prognostic value of muscle atrophy in cirrhosis using psoas muscle thickness on computed tomography	Sarcopenia and mortality	10.1016/J.JHEP.2014.02.026	95	J HEPATOL	2014
			LT				
			MELD score				
			Psoas muscle thickness (TPMT)				
			Prognostic marker				
8	VAN VUGT JLA	Systematic Review and Meta-analysis of the Impact of Computed Tomography–Assessed Skeletal Muscle Mass on Outcome in Patients Awaiting or Undergoing Liver Transplantation	Sarcopenia assessment	10.1111/AJT.13732	92	AM J TRANSPLANT	2016
			LT outcomes				
			MELD score supplementation				
			Muscle waste as a prognostic marker				
			Preoperative skeletal muscle mass and survival				
9	KAIDO T	Impact of Sarcopenia on Survival in Patients Undergoing Living Donor Liver Transplantation	Sarcopenia assessment	10.1111/AJT.12221	91	AM J TRANSPLANT	2013
			LDLT				
			Post-transplantation mortality				
			Muscle mass correlation				
			Perioperative nutritional therapy				
10	MONTANO-LOZA AJ	Inclusion of sarcopenia within MELD (MELD-Sarcopenia) and the prediction of mortality in patients with cirrhosis	Sarcopenia impact on LT	10.1038/CTG.2015.31	91	CLIN TRANSL GASTROEN	2015
			MELD score limitations				
			Muscularity assessment improvement				
			Mortality prediction in cirrhosis				
			Sarcopenia as a prognostic indicator				

Six of the top ten highly cited references were also among the top ten most co-cited references. Particularly, we found that ENGLESBE MJ (DOI: 10.1016/j.jamcollsurg.2010.03.039), MONTANO-LOZA AJ (DOI: 10.1016/j.cgh.2011.08.028), and TANDON P (DOI: 10.1002/lt.23495) not only ranked among the top three highly cited references but also ranked among the top three most co-cited references. Furthermore, we observed that MONTANO-LOZA AJ had the greatest impact, as three of the top ten most co-cited references were his works. In his 2014 article in “LT” (DOI: 10.1002/LT.23863), he presented a new and critical viewpoint: severe sarcopenia may not be linked to outcomes after LT (Table 4). However, all of these high-impact articles were clinical studies, and there is currently a lack of basic research exploring the underlying mechanisms between sarcopenia and LT.

### 3.6 Time zone analysis of authors and timeline analysis of references

**Figure 4:**
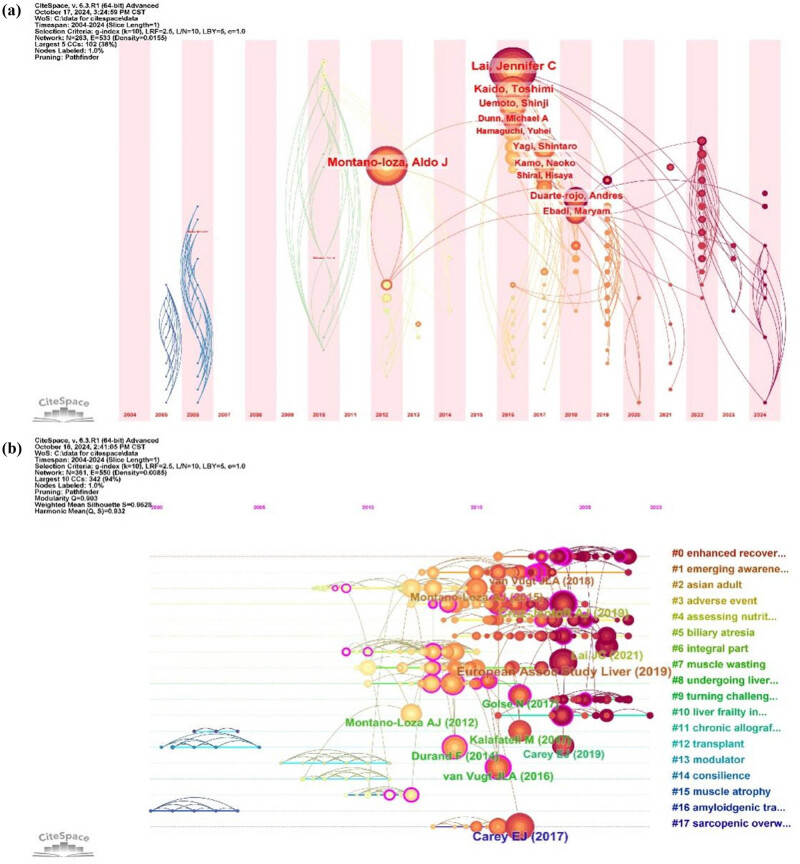
Visualized analysis of time zone and timeline. (a) Time zone map of authors on research of sarcopenia and LT. (b) Timeline map of references on research of sarcopenia and LT.

To gain a comprehensive understanding of the distribution of authors, collaboration networks, and the dynamic evolution of research hotspots in the fields of sarcopenia and LT, we performed a temporal analysis of the authors in this domain. The results revealed that representative authors can be categorized into three distinct periods. In the first period, Montano-Loza, Aldo J, emerged as a leading figure, driving the surge in publications in 2012. According to the author’s annual ring analysis, Montano-Loza, Aldo J not only published extensively but also achieved a high citation rate, maintaining significant scholarly attention in subsequent years. The second period, spanning from 2016 to 2019, was marked by a dramatic increase in publications, with key figures such as Lai, Jennifer C, and Kaido, Toshimi becoming prominent contributors. This phase witnessed the rise of numerous highly cited scholars, reflecting a shift toward a more diversified authorship landscape compared to the first period, where one prominent author stood out. Additionally, collaborations among various academic groups strengthened during this time, particularly within Asian academic circles, with Japan rapidly gaining prominence. In the following 2 years, the field experienced a temporary decline in research activity until the third phase began in 2022, when the research trend revived. During this phase, new scholars formed tight-knit collaborations, and citations continued to rise. However, the long-term significance and value of research conducted during this phase remain to be determined through future assessments (Figure 4a).

We conducted a more detailed global analysis of the evolving research hotspots in this field through a timeline graph. The results revealed that research in this domain has been particularly concentrated since 2012, forming 17 distinct thematic clusters (Figure 4b). Themes such as #0 Enhanced Recovery, #1 Emerging Awareness, #3 Adverse Event, #4 Assessing Nutrition, #7 Muscle Wasting, #11 Chronic Allograft, and #17 Sarcopenic Overweight have gained increasing attention over time, reflecting their growing prominence in the past decade. Classical LT-related topics, such as #8 Undergoing LT, #9 Turning Challenge, and #10 Liver Frailty Index, have continued to attract sustained attention. Notably, the most representative articles for each of these themes are among the top ten most-cited publications in the field.

### 3.7 Analysis of journals’ and co-cited journals’ distribution

**Figure 5:**
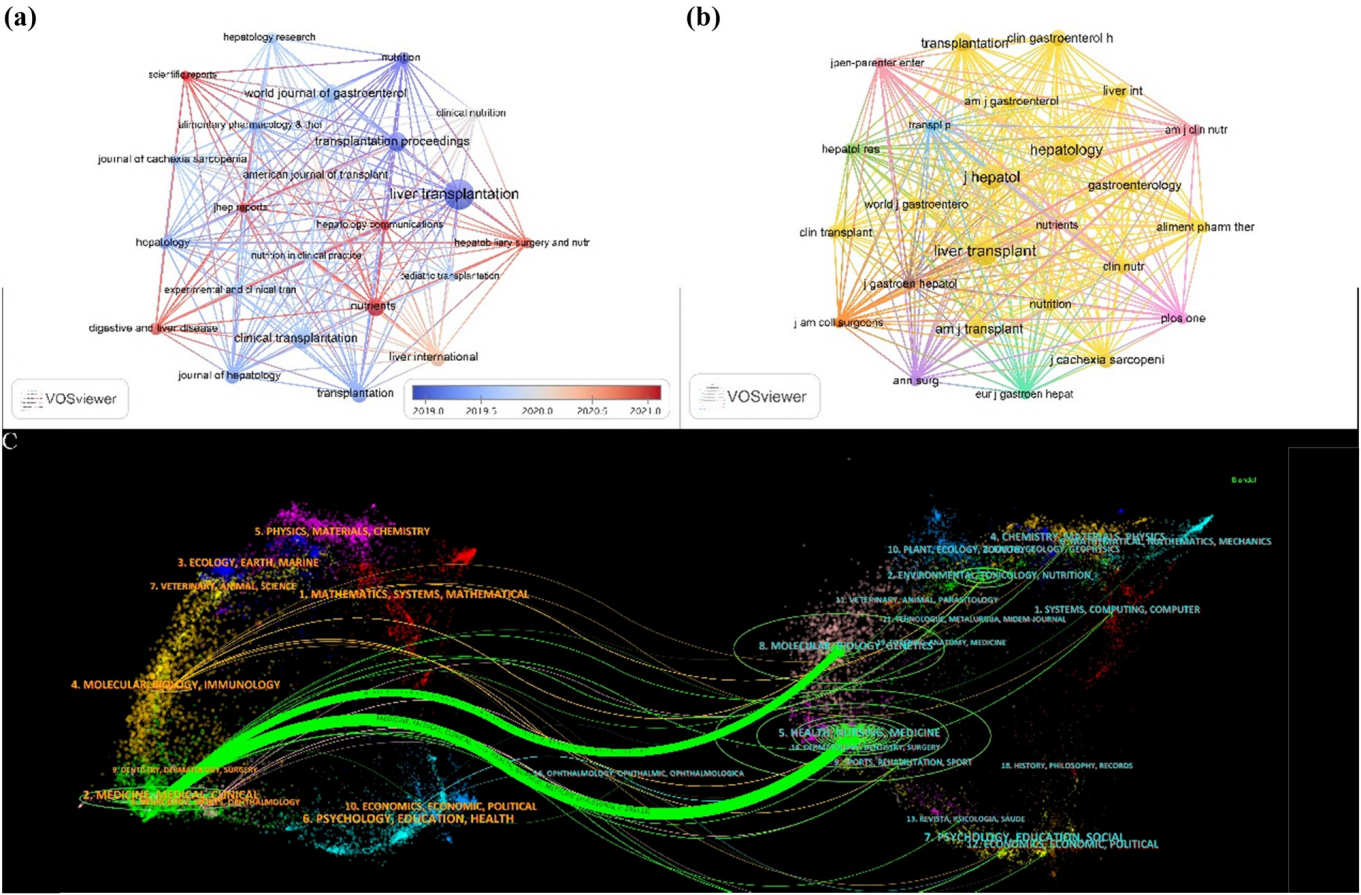
Network visualization of journals and co-cited journals. (a) Network map of journals on research of sarcopenia and LT. (b) Network map of co-cited journals on research of sarcopenia and LT. (c) Dual-map overlay of journals on research of sarcopenia and LT (left: clustering of citing journals; right: clustering of cited journals; colored path: citation relationships between the citing journals and the cited journals).

We performed a statistical analysis of 146 journals that published literature in the fields of sarcopenia and LT over the past two decades. Among these, *LT* had the highest publication volume (*n* = 38; 22.9%) and received the most citations (*n* = 3,039; 35.1%). Among the top ten highly cited journals, eight are ranked in the Q1 quartile of the Journal Citation Reports (JCR), indicating their high recognition and strong willingness to disseminate research in this field. All eight of these journals have an impact factor greater than four, with *Journal of Hepatology* having the highest impact factor (IF = 26.8). Its citation count ranked second only to *LT* (*n* = 1,599, 18.5%), with 12 publications (7.2%). Following closely was *Hepatology* (IF = 12.9), with 1,062 citations (12.3%) and 9 publications (5.4%). Although these two journals ranked eighth and tenth in terms of total publications, their average citations per publication ranked first (*n* = 133.25) and second (*n* = 118.00), respectively (Table 5). This suggests that these prestigious journals have rigorous manuscript selection processes and that the published articles possess high scientific value. These journals were closely interconnected, and based on the publication timeline, we can categorize them into four distinct periods:

(1) The 2019.0–2019.5 period, represented by *LT*, *Nutrition*, and *Transplantation Proceedings*.
(2) The 2019.5–2020.0 period, represented by *Clinical Transplantation* and *World Journal of Gastroenterology*.
(3) The 2020.0–2020.5 period, during which *Liver International* (IF = 6.0; Q1) and *American Journal of Transplantation* (IF = 8.9; Q1) led with high publication volumes.
(4) The 2020.5–2021.0 period, during which journals such as *Nutrients* (IF = 4.8, Q1) showed sustained growth in publication volume (Figure 5a).

**Table 5:** Top ten journals and co-cited journals on research of sarcopenia and LT

Rank	Journal	Documents	Citations	Average number of citations/publications	JIF (2024)	JCR	Co-cited Journal	Co-citations	JIF (2024)	JCR
1	Liver Transplantation	38	3,039	79.97	4.7	Q1	Journal of Liver Transplantation	1,806	4.7	Q1
2	Clinical Transplantation	20	402	20.10	1.9	Q3	Hepatology	1,380	12.9	Q1
3	Transplantation Proceedings	18	120	6.67	0.8	Q4	Journal of Hepatology	1,251	26.8	Q1
4	World Journal of Gastroenterology	17	684	40.24	4.3	Q1	American Journal of Transplantation	780	8.9	Q1
5	Nutrients	16	279	17.44	4.8	Q1	Transplantation	638	5.3	Q1
6	Liver International	13	272	20.92	6.0	Q1	Clinical Gastroenterology and Hepatology	495	11.6	Q1
7	Transplantation	13	501	38.54	5.3	Q1	Journal of Cachexia, Sarcopenia and Muscle	495	9.4	Q1
8	Journal of Hepatology	12	1,599	133.25	26.8	Q1	Gastroenterology	455	25.7	Q1
9	American Journal of Transplantation	10	707	70.70	8.9	Q1	Liver International	409	6.0	Q1
10	Hepatology	9	1,062	118.00	12.9	Q1	Nutrition	391	3.2	Q2

JIF, journal impact factor; JCR, journal citation reports.

Regarding co-cited journal analysis, nine out of the top ten journals were in the Q1 quartile, with only one in Q2. Four journals had an impact factor exceeding 10, and two surpassed 20. Notably, 50% of the journals exhibited a co-citation count greater than 500. Among these, *LT* led with the highest co-citation frequency (*n* = 1,806; 22.3%), followed by *Hepatology* (*n* = 1,380; 17.0%), *Journal of Hepatology* (*n* = 1,251; 15.4%), *American Journal of Transplantation* (*n* = 780; 9.6%), and *Transplantation* (*n* = 638; 7.9%) (Table 5). A co-citation network diagram further revealed robust, intricate, and tightly interconnected co-citation relationships among the top 10 journals (highlighted by yellow circles) (Figure 5b).

The dual-map overlay serves as a tool to visualize citation link mapping from the global citing base map to the global cited base map. Using this method, we analyzed the citation relationships between different journals and their distribution across various disciplines. As depicted in Figure 5c, two primary green citation paths represented the literature published in Molecular/Biology/Genetics (*z*-value = 1.85; *f*-value = 121,667) and Health/Nursing/Medicine, both predominantly cited by literature within the Medicine/Medical/Clinical field.

### 3.8 Analysis of keyword bursts

**Figure 6:**
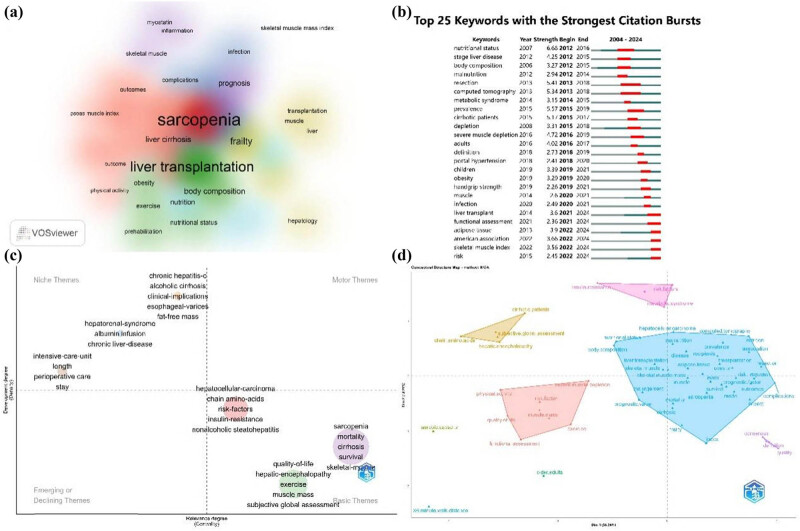
Visualized analysis of keyword bursts and thematic words. (a) Density visualization of co-occurrence author keywords of 448 literature reports. Changes in color refer to the clustering density (core areas within the research field). (b) Burst strength and time duration of the top 25 keywords with the strongest citation bursts. (c) Thematic analysis of the field of LT and sarcopenia. (Themes are grouped into four broad categories according to their development and relevance degrees: niche themes, motor themes, emerging or declining themes, and basic themes.) (d) Conceptual structure map of keyword burst.

To comprehensively and accurately identify the core themes, research trends, and emerging directions in the field, we conducted a co-occurrence analysis combining keywords and author keywords. The top 25 author keyword network was visualized using a density diagram. We observed that keywords centered around LT, along with physical condition-related terms (e.g., body composition, obesity, exercise, prehabilitation, and nutritional status) (green), emerged early and have remained a focus of sustained attention, representing classic themes in the field. In contrast, keywords centered around sarcopenia, along with outcome-related terms, liver cirrhosis, and physical evaluation (e.g., psoas muscle index and physical activity) (red), likely represent emerging research directions or hot topics. Furthermore, research related to complications and infections is relatively sparse (blue), while studies on frailty, hepatology, muscle, and similar topics are more abundant (yellow). Notably, keywords such as myostatin, inflammation, skeletal muscle, and prognosis not only frequently appeared in the literature but also emerged later (purple), indicating that these are recent research hotspots (Figure 6a).

Additionally, we analyzed the burst terms among the top 25 based on their emergence time and citation intensity. Nutritional status was identified as the earliest burst term, with the strongest citation bursts between 2012 and 2016 (Str = 6.68). This was followed by surges in research on resection (Str = 5.41) and computed tomography (Str = 5.34) during the 2013–2018 period. Emerging hotspot themes in the field began to surface during the mid-period of the 2004–2024 period, with the overall trend shifting from the clinical/nutritional status to muscle depletion/complications and eventually toward more refined sarcopenia evaluation standards (functional assessment and skeletal muscle index – SMI) (Figure 6b).

### 3.9 Analysis of hotpots and frontiers

We employed thematic term analysis to explore the core themes in the field of sarcopenia combined with LT. By analyzing the development and relevance degree of the terms, they were categorized into four domains: niche themes, basic themes, emerging or declining themes, and motor themes. In niche fields, themes focused on medical care (including intensive care unit length and perioperative care stay), liver diseases (e.g., hepatorenal syndrome, albumin infusion, and chronic liver disease), and protopathy and complications (e.g., chronic hepatitis C, alcoholic cirrhosis, clinical implications, esophageal varices, and fat-free mass). In classical and well-developed fields, themes were clustered into three groups: underlying mechanism (e.g., hepatocellular carcinoma, chain amino acids, risk factors, insulin resistance, and nonalcoholic steatohepatitis), assessment and intervention (e.g., quality of life, hepatic encephalopathy, exercise, muscle mass, and subjective global assessment), and epidemiology and prognosis (e.g., sarcopenia, mortality, cirrhosis, survival, and skeletal muscle) (Figure 6c). However, no themes were identified in the motor and emerging/declining fields, suggesting that these two areas may represent breakthrough opportunities for future research.

Additionally, a descending dimension method with the MCA algorithm was used to perform a re-clustering analysis of co-occurrence keywords. The resulting conceptual structure map revealed five clusters. The largest cluster, shown in blue, encompassed the majority of themes, focusing on clinical nutrition assessment, complications, transplantation, and prognosis (Figure 6d). These results are consistent with the previous analysis and further expand the understanding of the hot topics in this field from another perspective.

**Figure 7:**
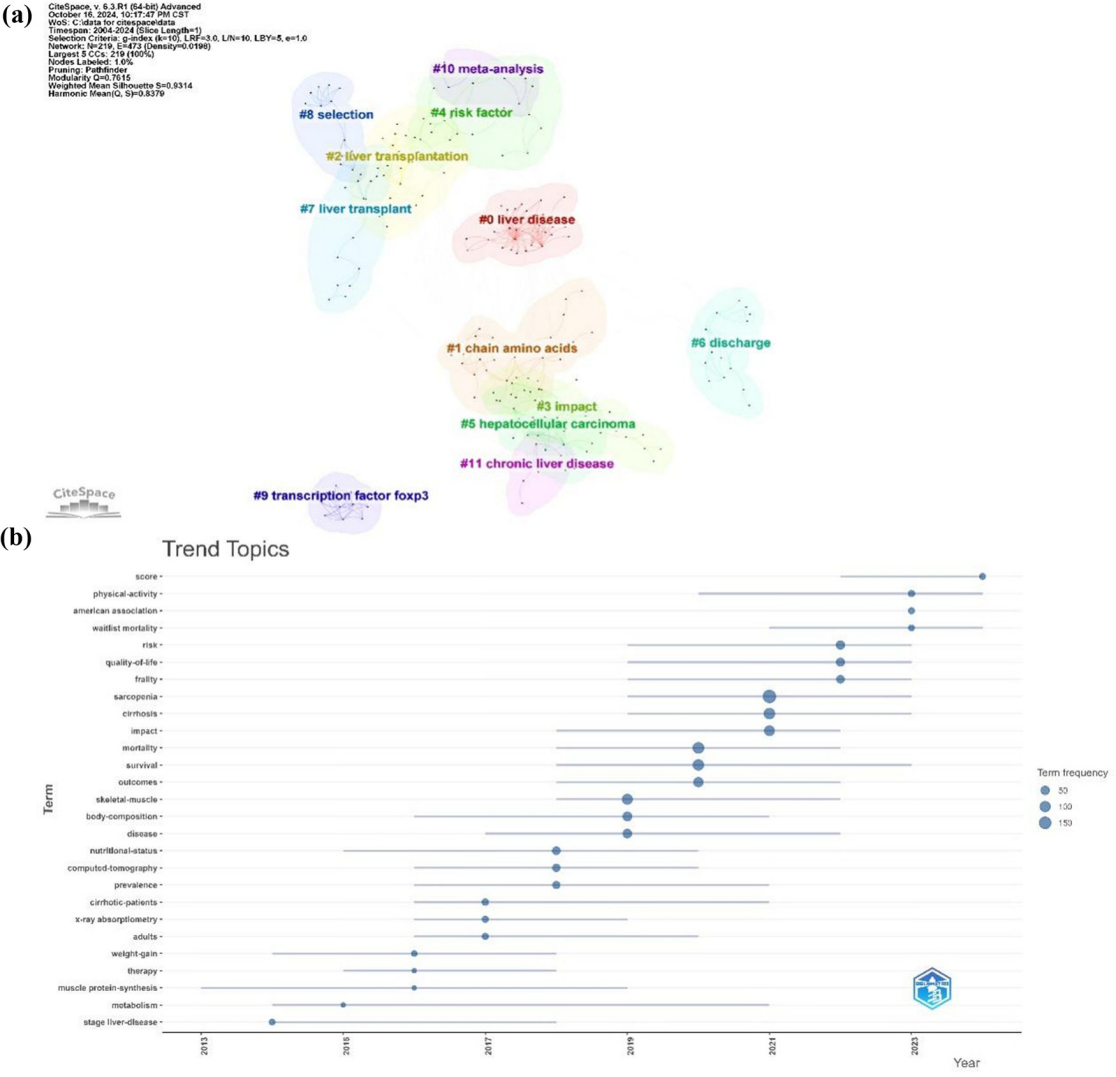
Trend analysis of research on LT and sarcopenia. (a) Clustering analysis of keywords. (b) Timeline of topic trends in the research refers to LT and sarcopenia.

Furthermore, CiteSpace was used to conduct a clustering analysis of the keywords in the literature from 2004 to 2024 in this field. A total of 12 clustering themes were obtained, among which #3 impact, #6 discharge, #8 selection, #9 transcription factor foxp3, and #10 meta-analysis are new themes differing from previous analyses (Figure 7a), indicating new directions for exploration. Simultaneously, we conducted a temporal trend visualization analysis of trend topics (Figure 7b). The results are largely consistent with Figure 6b. It is particularly noteworthy that the nine topics during the period from 2019 to 2021, including sarcopenia, cirrhosis, impact, mortality, survival, outcomes, skeletal muscle, body composition, and disease, received the greatest attention and emphasis, with rapid development in research, literature, collaboration, and citations surrounding them. Overall, the focus of the topics has shifted from pathophysiology (e.g., stage liver disease, metabolism, etc.) to the current refined evaluation criteria and prognostic models (e.g., score, waitlist mortality, etc.). The shift in research hotspots indicates a change in scientists’ attitudes and approaches to research in this field, pointing to potential rapidly developing research directions and key areas in the future.

### 3.10 Analysis of biological mechanisms underlying LT and sarcopenia

**Figure 8:**
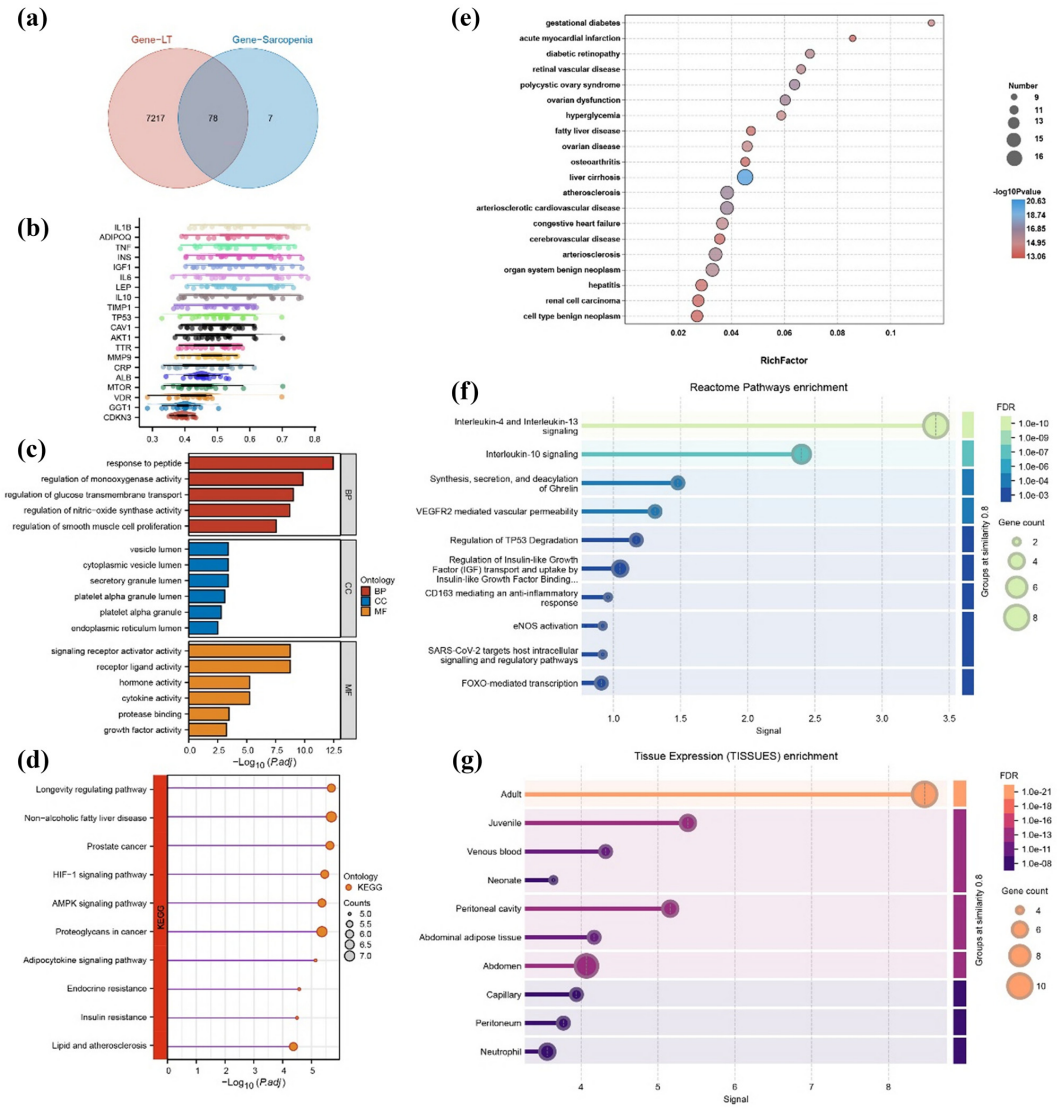
Biological and genetic analyses of LT and sarcopenia. (a) Potential interactive target genes for LT and sarcopenia. (b) Friends analysis of the target genes. (c) GO analysis of the target genes. (d) KEGG analysis of the target genes. (e) DO analysis of the target genes. (f) Reactome pathway enrichment analysis of the target genes. (g) TISSUES analysis of the targets. GO: gene ontology; KEGG: Kyoto Encyclopedia of Genes and Genomes; DO: disease ontology; TISSUES: tissue expression. *P <0.05, **P <0.01, and ***P <0.001.

According to the screening criteria, we retrieved 7,295 LT-related target genes and 85 sarcopenia-related target genes from the GeneCards database. By intersecting these two datasets, we identified 78 overlapping target genes that are commonly related to both LT and sarcopenia (Figure 8a). Due to the lack of gene expression profile data, we used Friends analysis as an alternative to machine learning for further screening of hub genes that potentially play key roles in the BPs of these two disease phenotypes. As shown in the raincloud plot, the top 20 out of 78 biphenotypic genes were ranked based on gene similarity, with IL1B showing the strongest correlation with other genes (similarity index (SI) = 0.60), suggesting its potential central role. Other closely related genes included ADIPOQ, TNF, INS, IGF1, IL6, LEP, IL10, TIMP1, TP53, and CAV1, all of which demonstrated strong correlations with SI values greater than 0.5, indicating their collaborative involvement in the BPs underlying LT and sarcopenia (Figure 8b).

To explore the potential biological mechanisms linking these two disease phenotypes, we conducted GO and KEGG enrichment analyses on the top 20 hub genes. In terms of BPs, pathways such as “response to peptide,” “regulation of monooxygenase activity,” “regulation of glucose transmembrane transport,” “regulation of nitric-oxide synthase activity,” and “regulation of smooth muscle cell proliferation” (all *P*.adj <0.001) were identified as the primary mechanisms involved in the progression of these diseases. CC analysis highlighted terms like “vesicle lumen,” “cytoplasmic vesicle lumen,” “secretory granule lumen,” “platelet alpha granule lumen,” “platelet alpha granule,” and “endoplasmic reticulum lumen” (all *P*.adj <0.01). MF analysis revealed functions such as “signaling receptor activator activity,” “receptor ligand activity,” “hormone activity,” “cytokine activity,” “protease binding,” and “growth factor activity” (all *P*.adj <0.001) as being closely related to these two disease phenotypes (Figure 8c). Based on KEGG pathway analysis, the key pathways primarily focused on cellular energy metabolism (e.g., AMPK signaling pathway), lipid metabolism (e.g., adipocytokine signaling pathway), endocrine metabolism (e.g., endocrine resistance), and tumor metabolism (e.g., proteoglycans in cancer) (all *P*.adj <0.001) (Figure 8d).

Furthermore, leveraging the disease association information of these hub genes, we constructed a disease association network. This revealed that liver diseases (e.g., fatty liver disease, liver cirrhosis), diabetes-related diseases (e.g., diabetic retinopathy, gestational diabetes), cardiovascular diseases (e.g., atherosclerotic cardiovascular disease, acute vascular disease, arteriosclerotic cardiovascular disease), and tumor-related diseases (e.g., organ system benign neoplasm and renal cell carcinoma) were significantly associated with LT and sarcopenia (all *P*.adj <0.001). These findings suggest the existence of common features and potential shared mechanisms among these diseases (Figure 8e).

To further elucidate the potential biomolecular mechanisms and histological distribution of these top 20 hub genes, we performed reactome pathway analysis and tissue expression (TISSUES) enrichment analyses. Beyond confirming the regulation of insulin-like growth factor (IGF) consistent with the aforementioned enrichment results, we uncovered additional precise pathways: interleukin-4/interleukin-13/interleukin-10 signaling, ghrelin BP, VEGFR2-mediated vascular permeability, regulation of TP53 degradation, CD163-mediated anti-inflammatory response, eNOS (endothelial nitric-oxide synthase) activation, SARS-CoV-2 targeting host intracellular signaling and regulatory pathways, and FOXO-mediated transcription (Figure 8f). Additionally, we found that these hub genes were predominantly enriched in adults compared to minors and infants and exhibited high expression levels in abdominal adipose tissues, the peritoneum, venous blood, capillaries, and neutrophils (Figure 8g).

## 4 Discussion

Sarcopenia is an age-related condition that has garnered increasing international attention [31,32]. It is closely linked to nutrition [33], liver diseases [34], cardiovascular diseases [35], and cancers [36]. In our country, sarcopenia significantly affects the survival and quality of life of patients undergoing LT for end-stage liver disease [37,38]. Both pre- and post-transplantation sarcopenia have been reported to correlate with poor postoperative outcomes, emerging as a critical research frontier. Therefore, elucidating the underlying mechanisms and interactions between sarcopenia and LT is crucial. This study innovatively integrated bibliometric analysis with bioinformatics tools, aiming to move beyond previous research that predominantly focused on surgical outcomes or mechanisms of muscle atrophy [39,40], thereby providing a multidimensional perspective on the knowledge structure, evolutionary trajectory, and future trends of this emerging field. In contrast to previous studies predominantly focused on validating prognostic associations or isolated mechanisms, the core value of this research lies in constructing a multi-level analytical framework of “macro-trends–knowledge structure–molecular network.” It organically links active research clusters, key scholarly contributions, potential driver genes, and biological pathways for the first time, thereby providing an unprecedented systematic perspective and a series of testable new hypotheses for understanding the pathogenesis of sarcopenia in the context of LT.

We identified 448 studies related to LT and sarcopenia over the past 20 years from WoSCC. Mathematical modeling of publication trends revealed a marked exponential growth in research output beginning in 2014 (Figure 2a), a pattern indicative of the field’s growing significance and momentum. This surge coincides with the initial operationalization of sarcopenia by the European Working Group on Sarcopenia in Older People (EWGSOP), which provided essential diagnostic criteria that galvanized systematic research [8]. Prior to this consensus, studies primarily emphasized the role of nutritional indicators – such as albumin and body composition – in LT prognosis [41], yet lacked standardized, objective measures for assessing sarcopenia. The establishment of consensus definitions enabled skeletal muscle evaluation to evolve into a widely adopted clinical tool for nutritional assessment, disease stratification, and prognostic prediction. Furthermore, the accelerated growth since 2014 likely reflects increasing recognition of sarcopenia as a pivotal determinant of post-LT outcomes and quality of life. Broader trends, including heightened focus on nutritional health during global public health challenges such as the COVID-19 pandemic, influenza, and monkeypox [42], may have also contributed to the expanded scientific interest in this area.

An analysis of research performance across countries and regions reveals significant geographic disparities and an evolution in collaborative patterns within the field of LT and sarcopenia research. European and North American countries dominate in both academic output (*n* = 309; 69.0%) and influence (*n* = 15,606; 64.4%), while Asian countries follow distantly (133 publications, 29.7%; 3,826 citations, 15.8%) This distribution strongly correlates with levels of economic development, healthcare infrastructure, and research investment, underscoring the role of high-resource settings in shaping global knowledge production in this domain. Notably, despite an overall increase in international collaboration, substantial barriers to cooperation persist among leading institutions. For instance, while Kyoto University in Japan is recognized as a top-contributing institution, it exhibits limited collaborative links with key North American and European research entities (Figure 3b). This relative isolation may stem from several factors: differences in study populations and disease etiology may lead to divergent research priorities; the lack of standardized diagnostic criteria may hinder comparative studies; and there may be lingering challenges in the visibility and integration of non-English speaking research communities within global academic networks. These observations highlight the need for standardized methodologies and shared data platforms to improve the generalizability and interoperability of research findings.

Furthermore, author collaboration networks reveal that some highly productive Japanese research teams exhibit a pattern of high output yet relatively limited international engagement. Their early and prolific contributions (Figure 3c) were characterized predominantly by domestic or institutional partnerships. This insular collaboration pattern may be influenced by region-specific study designs – such as a focus on single-center cohorts or Asian patient populations – as well as distinct clinical priorities. While such an approach fosters regional research identity and may address local health concerns, it may also limit the global dissemination and integration of findings, as reflected in the distinct cluster structures observed in co-citation networks. Temporal analysis indicates an encouraging trend toward diversification since 2019, with the emergence of new countries, institutions, and researchers entering the field. However, achieving truly equitable and deeply integrated global research collaboration remains challenging. Future efforts should prioritize the development of inclusive partnerships that account for varied regional disease burdens and clinical needs. Strategic resource allocation and policy support should aim to foster methodologically robust, translatable, and equitable research partnerships that can address both global priorities and locally relevant questions in LT and sarcopenia.

The three most cited and co-cited references are identical, underscoring their foundational role in this field. The study by Englesbe et al. was among the first to systematically establish a robust correlation between sarcopenia and post-LT mortality, proposing sarcopenia as an objective marker of patient frailty with potential implications for clinical decision-making and organ allocation policies [43]. Montano-loza et al. further articulated the prognostic impact of muscle wasting in patients with cirrhosis, stimulating subsequent research into mechanisms, diagnostics, and interventions in this subpopulation [44]. Tandon et al. provided the first systematic assessment of sarcopenia prevalence among patients on the LT waitlist and demonstrated its independent prognostic value superior to conventional nutritional metrics such as BMI [45]. These three highly cited/co-cited references established the foundation for sarcopenia research in LT, triggering a wave of related studies. Furthermore, dual-map analysis reveals that research themes on LT and sarcopenia have evolved from an initial focus on pharmacological and clinical aspects toward molecular mechanisms, public health, and integrated therapeutic strategies (Figure 5c). This shift emphasizes the growing importance of multidisciplinary approaches and suggests a future research trajectory increasingly oriented toward mechanistic insight and translational applications.

In CiteSpace, modularity (*Q*-index) and weighted mean silhouettes (*S*-index) are used to evaluate the overall framework performance of keyword clustering networks. A *Q*-value >0.3 is regarded as an indication of a strong network structure. The credibility threshold is set as follows: an *S*-value >0.5 is considered moderate and reasonable and an *S*-value >0.7 is highly credible [46]. In our study, the *Q*-value was 0.7615 and the *S*-value was 0.9314, suggesting a robust and highly credible clustering network (Figure 7a). This network illustrated that research in the field primarily focused on four aspects: liver diseases, tumors, risk factors and prognosis, and molecular mechanisms. Early studies primarily centered on the prevalence of sarcopenia and its impact on the prognosis of LT patients. Carey et al. defined sarcopenia in patients with end-stage liver disease through a multicenter study and explored its prognostic implications [47]. Golse et al. proposed a new definition of sarcopenia in LT patients and suggested diagnostic criteria based on CT imaging [48]. As research advanced, scholars began to investigate the pathogenesis of sarcopenia, including hepatic–muscle axis mediators (e.g., hyperammonemia, low growth hormone levels, endotoxemia, etc.) [49–51], as well as the role of gut microbiota [52]. In recent years, research has gradually shifted toward clinical interventions such as nutritional support, resistance training, and hormone supplementation to improve the prognosis of sarcopenic patients [53]. Current research hotpots and trends have shifted from a broad form to a more detailed one. The proposal of novel scoring models, such as the MELD-sarcopenia scoring model [54] and sarco-model (MELD-Na + sarcopenia) [55], scientifically underscored the significance of incorporating sarcopenia into LT evaluation systems and clinical decision-making for liver allocation. Bi et al. explored the application of artificial intelligence in cancer imaging, offering new insights for the radiological assessment of sarcopenia [56]. Kim et al. developed a machine learning model for sarcopenia identification based on muscle radiomics features [57]. Additionally, Chinese researchers have made unique contributions to LT for hepatocellular carcinoma (HCC), with Xiao et al. identifying chitinase-3-like protein 1 (CHI3L1) as a biomarker associated with both sarcopenia and tumor recurrence risk [58,59]. They also proposed using radiomics to assess the degree of sarcopenia in HCC patients [60]. Future research will increasingly focus on the precise, diversified, and intelligent assessment of sarcopenia, integrating radiomics and artificial intelligence technologies to further optimize prognostic prediction models. The research paradigm in this field is undergoing a profound shift from “phenomenological observation” to “mechanistic dissection” and finally to “precision intervention.”

Currently, the mechanistic research on sarcopenia in the domain of LT is still in its preliminary stages. Inter-organ interactions have become a novel research focus. Studies suggest that the gut–liver–muscle axis mediates a multi-organ interaction, which is a critical factor in the onset and progression of sarcopenia. The specific mechanisms include: (1) impaired liver function leading to hyperammonemia, which inhibits muscle synthesis, activates muscle autophagy, and enhances the ubiquitin–proteasome pathway, thereby accelerating muscle breakdown [49]. (2) Low levels of growth hormone resulting in reduced muscle protein synthesis, further exacerbating sarcopenia [50]. (3) Cirrhotic patients frequently endure gut microbiota dysbiosis and endotoxemia, where the former indirectly affects muscle metabolism through modulation of gut barrier function and immune regulation, while the latter activates inflammatory pathways that inhibit muscle synthesis and promote muscle breakdown [51,52]. (4) Fibroblast growth factor 21 (FGF21) may influence muscle mass by regulating mitochondrial function and energy metabolism [61]. Although these studies have revealed some of the mechanisms of sarcopenia in LT, the overall mechanism remains ambiguous. Our bioinformatics findings push this understanding to a broader dimension. The 78 dual-phenotype target genes and 20 hub genes (e.g., IL1B, ADIPOQ, etc.) we identified do not function in isolation but are intensively enriched in several highly interconnected functional modules: 1) The Immunometabolism Cross-Talk Network: the intertwining of insulin resistance and anti-inflammatory response pathways mediated by IL-4/IL-10/IL-13 suggests that the chronic inflammatory microenvironment may directly disrupt muscle homeostasis by modulating insulin sensitivity and lipid metabolism (ADIPOQ function). This provides a novel mechanism for understanding the link between cirrhosis-associated metabolic disorders and sarcopenia. 2) The Cellular Stress and Survival Regulation Network: pathways such as TP53, FOXO, AMPK, and HIF-1 constitute a core regulatory module responding to energy crisis, oxidative stress, and DNA damage. We hypothesize that various perioperative stressors in LT (ischemia–reperfusion injury, metabolic disorders, and infection [6]) may synergistically activate this network, inducing mitochondrial dysfunction and protein degradation, thereby exacerbating or triggering muscle wasting. This moves beyond the traditional nutritional explanation framework, positioning sarcopenia as a manifestation of a systemic stress response in muscle tissues. 3) Potential Inter-organ Communication Mechanisms: Factors like FGF21 may not only be metabolically active hormones secreted by the liver but also key messengers in inter-organ crosstalk. These findings collectively paint a picture far more complex than the “liver–muscle axis,” hinting at a “multi-organ network disease” model involving the liver, gut, immune system, adipose tissues, and muscles. This lays a solid theoretical foundation for developing future combination interventions targeting specific pathways (e.g., modulating FOXO activity, improving insulin sensitivity, or targeting specific inflammatory factors). Furthermore, our study also raised a key scientific question: Does the predominant mechanism of sarcopenia vary based on its etiology and population? In HCC-associated sarcopenia, activation of the TP53 pathway might simultaneously drive carcinogenesis and muscle atrophy, forming a “comorbid” mechanism. While the role of CHI3L1 [59,60] suggested a previously unrecognized interaction between the tumor microenvironment and muscle metabolism. Therefore, future research must emphasize population specificity and develop precise phenotyping, rather than treating sarcopenia as a single disease.

Looking forward, this study points to several highly promising directions: first, leveraging multi-omics data integration to further elucidate the dynamic expression changes of these hub genes before and after LT and their causal relationship with clinical outcomes. Second, exploring the integration of radiomics and biomarkers could help build a “digital biopsy” predictive model that reflects both macro-morphology and micro-function. Third, promoting mechanism-oriented clinical trials: our findings strongly suggest that future interventions should not be limited to nutritional support or exercise alone but should consider testing the effectiveness of combination therapies targeting immunometabolism (e.g., anti-inflammatory nutritional formulations, insulin sensitizers) or modulating cellular stress (e.g., AMPK activators) in the LT perioperative period.

Certainly, recognizing the limitations of the study is critical for determining future research objectives. One significant issue is the reliance on retrospective data from a single database, which may not include the entire corpus of literature on LT and sarcopenia (selection bias). Second, the results of this study may be influenced by publication bias (i.e., studies with positive results are more likely to be published). Furthermore, the mechanisms inferred from bioinformatics require functional validation and causal confirmation through *in vitro* cell models, genetically modified animal experiments, and prospective clinical studies. Future research should concentrate on multi-center collaborations involving diverse patient populations to enhance the generalizability of findings. Moreover, prospective studies are required to investigate the temporal dynamics of muscle mass changes in LT patients and their implications on recovery and survival, filling the major gaps revealed in our research.

## 5 Conclusions

This work integrates bibliometric and bioinformatics approaches to comprehensively evaluate the literature on LT and sarcopenia, uncovering the key research trends, shared biomarkers, and potential pathological processes. Both illnesses share numerous similarities in etiology and progression but may also engage in reciprocal aggravation, forming a complex vicious cycle. Nevertheless, high-quality evidence elucidating their mechanistic interplay remains limited. Moving forward, research should prioritize the convergence of mechanistic exploration and clinical translation, which will be essential to advancing the field and improving prognostic outcomes for affected patients.

## References

[1] Samuel D, De Martin E, Berg T, Berenguer M, Burra P, Fondevila C, et al. EASL clinical practice guidelines on liver transplantation. J Hepatol. 2024;81(6):1040–86. 10.1016/j.jhep.2024.07.032 39487043

[2] Olivo R, Guarrera JV, Pyrsopoulos NT. Liver transplantation for acute liver failure. Clin Liver Dis. 2018;22(2):409–17. 10.1016/j.cld.2018.01.014 29605075

[3] Kohli R, Cortes M, Heaton ND, Dhawan A. Liver transplantation in children: state of the art and future perspectives. Arch Dis Child. 2018;103(2):192–8. 10.1136/archdischild-2015-310023 28918383

[4] Sugawara Y, Hibi T. Recent trends and new developments in liver transplantation. Biosci Trends. 2024;18(3):206–11. 10.5582/bst.2024.01176 38945855

[5] Honerkamp I, Sandmann L, Richter N, Manns MP, Voigtländer T, Vondran FWR, et al. Surgical procedures in patients awaiting liver transplantation: Complications and impact on the liver function. J Clin Exp Hepatol. 2022;12(1):68–79. 10.1016/j.jceh.2021.03.011 PMC8766540 35068787

[6] Vidot H, Kline K, Cheng R, Finegan L, Lin A, Kempler E, et al. The relationship of obesity, nutritional status and muscle wasting in patients assessed for liver transplantation. Nutrients. 2019;11(9):2097. 10.3390/nu11092097 PMC6769900 31487854

[7] Fukuhara S, Kobayashi T, Hamaoka M, Naruhiko H, Oishi K, Namba Y, et al. Sarcopenia’s impact defined by grip strength and muscle mass on post-hepatectomy outcomes: A multicenter analysis. Vivo. 2024;38(6):2827–35. 10.21873/invivo.13763 PMC11535901 39477404

[8] Cruz-Jentoft AJ, Bahat G, Bauer J, Boirie Y, Bruyère O, Cederholm T, et al. Sarcopenia: Revised European consensus on definition and diagnosis. Age Ageing. 2019;48(1):16–31. 10.1093/ageing/afy169 PMC6322506 30312372

[9] Shimura Y, Kuramitsu K, Kido M, Komatsu S, Gon H, Fukushima K, et al. Factors predicting over-time weight increase after liver transplantation: A retrospective study. Transpl Proc. 2023;55(4):924–9. 10.1016/j.transproceed.2023.03.045 37095008

[10] Wiedmer P, Jung T, Castro JP, Pomatto LCD, Sun PY, Davies KJA, et al. Sarcopenia - Molecular mechanisms and open questions. Ageing Res Rev. 2021;65:101200. 10.1016/j.arr.2020.101200 33130247

[11] Eguchi Y, Suzuki M, Yamanaka H, Tamai H, Kobayashi T, Orita S, et al. Influence of skeletal muscle mass and spinal alignment on surgical outcomes for lumbar spinal stenosis. Asian Spine J. 2018;12(3):556–62. 10.4184/asj.2018.12.3.556 PMC6002163 29879785

[12] Jeong D, Lee SW, Jang HY, Kwon HM, Shin WJ, Song IK. Preoperative low muscle mass and early postoperative outcomes in children undergoing living donor liver transplantation: A retrospective study. Liver Transpl. 2024;30(1):83–93. 10.1097/LVT.0000000000000230 37526584

[13] Farquhar R, Matthews S, Baxter N, Rayers G, Ratnayake CBB, Robertson FP, et al. Sarcopenia and sarcopenic obesity on body composition analysis is a significant predictor of mortality in severe acute pancreatitis: A longitudinal observational study. World J Surg. 2023;47(11):2825–33. 10.1007/s00268-023-07122-1 PMC10545625 37541981

[14] Ulugerger Avci G, Bektan Kanat B, Can G, Suzan V, Unal D, Degirmenci P, et al. The impact of sarcopenia and obesity on mortality of older adults: Five years results. Ir J Med Sci. 2023;192(5):2209–16. 10.1007/s11845-023-03392-9 37202585

[15] Yu DG, Xu L. Impact evaluations of articles in current drug delivery based on web of science. Curr Drug Deliv. 2024;21(3):360–7. 10.2174/1567201820666230508115356 37157193

[16] Giménez-Espert MDC, Prado-Gascó VJ. Bibliometric analysis of six nursing journals from the Web of Science, 2012–2017. J Adv Nurs. 2019;75(3):543–54. 10.1111/jan.13868 30289557

[17] He T, Zou J, Sun K, Yang J. Global research status and frontiers on autophagy in hepatocellular carcinoma: a comprehensive bibliometric and visualized analysis. Int J Surg. 2024;110(5):2788–802. 10.1097/JS9.0000000000001202 PMC11093451 38376850

[18] Jiang T, Lin T, Shu X, Song Q, Dai M, Zhao Y, et al. Prevalence and prognostic value of preexisting sarcopenia in patients with mechanical ventilation: A systematic review and meta-analysis. Crit Care. 2022;26(1):140. 10.1186/s13054-022-04015-y PMC9109453 35578299

[19] Lin T, Dai M, Xu P, Sun L, Shu X, Xia X, et al. Prevalence of sarcopenia in pain patients and correlation between the two conditions: A systematic review and meta-analysis. J Am Med Dir Assoc. 2022;23(5):902.e1–.e20. 10.1016/j.jamda.2022.02.005 35339458

[20] Li J, Wu G, Zhang Y, Shi W. Optimizing flood predictions by integrating LSTM and physical-based models with mixed historical and simulated data. Heliyon. 2024;10(13):e33669. 10.1016/j.heliyon.2024.e33669 PMC11262550 39040386

[21] Vivaudou M. eeFit: A microsoft excel-embedded program for interactive analysis and fitting of experimental dose-response data. Biotechniques. 2019;66(4):186–93. 10.2144/btn-2018-0136 30987445

[22] Zhang L, Zheng H, Jiang ST, Liu YG, Zhang T, Zhang JW, et al. Worldwide research trends on tumor burden and immunotherapy: A bibliometric analysis. Int J Surg. 2024;110(3):1699–710. 10.1097/JS9.0000000000001022 PMC10942200 38181123

[23] Wang J, Shahzad F. A visualized and scientometric analysis of health literacy research. Front Public Health. 2021;9:811707. 10.3389/fpubh.2021.811707 PMC8830295 35155357

[24] Liaqat W, Altaf MT, Barutçular C, Zayed EM, Hussain T. Drought and sorghum: A bibliometric analysis using VOS viewer. J Biomol Struct Dyn. 2024;42(22):12317–29. 10.1080/07391102.2023.2269279 37837436

[25] Ye L, Liang R, Liu X, Li J, Yue J, Zhang X. Frailty and sarcopenia: A bibliometric analysis of their association and potential targets for intervention. Ageing Res Rev. 2023;92:102111. 10.1016/j.arr.2023.102111 38031836

[26] Zhou L, Min Y, Cao Q, Tan X, Cui Y, Wang J. Comprehensive analysis of the value of angiogenesis and stemness-related genes in the prognosis and immunotherapy of ovarian cancer. Biofactors. 2025;51(1):e2155. 10.1002/biof.2155 PMC11659921 39704033

[27] Dai L, Mugaanyi J, Cai X, Lu C, Lu C. Pancreatic adenocarcinoma associated immune-gene signature as a novo risk factor for clinical prognosis prediction in hepatocellular carcinoma. Sci Rep. 2022;12(1):11944. 10.1038/s41598-022-16155-w PMC9279485 35831362

[28] Schriml LM, Munro JB, Schor M, Olley D, McCracken C, Felix V, et al. The human disease ontology 2022 update. Nucleic acids Res. 2022;50(D1):D1255–d61. 10.1093/nar/gkab1063 PMC8728220 34755882

[29] Szklarczyk D, Kirsch R, Koutrouli M, Nastou K, Mehryary F, Hachilif R, et al. The STRING database in 2023: Protein-protein association networks and functional enrichment analyses for any sequenced genome of interest. Nucleic acids Res. 2023;51(D1):D638–d46. 10.1093/nar/gkac1000 PMC9825434 36370105

[30] Milacic M, Beavers D, Conley P, Gong C, Gillespie M, Griss J, et al. The reactome pathway knowledgebase 2024. Nucleic Acids Res. 2024;52(D1):D672–d8. 10.1093/nar/gkad1025 PMC10767911 37941124

[31] Wakabayashi H. Hospital-associated sarcopenia, acute sarcopenia, and iatrogenic sarcopenia: Prevention of sarcopenia during hospitalization. J Gen Fam Med. 2023;24(3):146–7. 10.1002/jgf2.625 PMC10227739 37261047

[32] Kuzuya M. Drug-related sarcopenia as a secondary sarcopenia. Geriatr Gerontol Int. 2024;24(2):195–203. 10.1111/ggi.14770 PMC11503558 38158766

[33] Tırnova İ, Gasimova M, Akay H, Sarıtürk Ç, Güven Mert A, Yenidünya Ö, et al. Low skeletal muscle mass as a proxy marker of sarcopenia is a risk factor for major complications in older patients undergoing curative colon resections for colon cancer. Front Med (Lausanne). 2024;11:1464978. 10.3389/fmed.2024.1464978 PMC11754278 39850105

[34] Saeki C, Tsubota A. Influencing factors and molecular pathogenesis of sarcopenia and osteosarcopenia in chronic liver disease. Life (Basel). 2021;11(9):899. 10.3390/life11090899 PMC8468289 34575048

[35] Damluji AA, Alfaraidhy M, AlHajri N, Rohant NN, Kumar M, Al Malouf C, et al. Sarcopenia and cardiovascular diseases. Circulation. 2023;147(20):1534–53. 10.1161/CIRCULATIONAHA.123.064071 PMC10180053 37186680

[36] Meza-Valderrama D, Marco E, Dávalos-Yerovi V, Muns MD, Tejero-Sánchez M, Duarte E, et al. Sarcopenia, malnutrition, and cachexia: Adapting definitions and terminology of nutritional disorders in older people with cancer. Nutrients. 2021;13(3):761. 10.3390/nu13030761 PMC7996854 33652812

[37] Campos-Varela I, Castells L, Quiroga S, Vargas V, Simon-Talero M. Frailty and sarcopenia in patients with acute-on-chronic liver failure: Assessment and risk in the liver transplant setting. Ann Hepatol. 2024;29(5):101515. 10.1016/j.aohep.2024.101515 38851394

[38] Zhou D, Zhang D, Zeng C, Zhang L, Gao X, Wang X. Impact of sarcopenia on the survival of patients undergoing liver transplantation for decompensated liver cirrhosis. J Cachexia Sarcopenia Muscle. 2023;14(6):2602–12. 10.1002/jcsm.13334 PMC10751414 37735907

[39] Wahlen BM, Mekkodathil A, Al-Thani H, El-Menyar A. Impact of sarcopenia in trauma and surgical patient population: A literature review. Asian J Surg. 2020;43(6):647–53. 10.1016/j.asjsur.2019.10.010 31796260

[40] Wilkinson DJ, Piasecki M, Atherton PJ. The age-related loss of skeletal muscle mass and function: Measurement and physiology of muscle fibre atrophy and muscle fibre loss in humans. Ageing Res Rev. 2018;47:123–32. 10.1016/j.arr.2018.07.005 PMC6202460 30048806

[41] Fujii W, Wada H, Hasegawa S, Mukai Y, Asukai K, Akita H, et al. Clinical impact of body composition on postoperative outcomes during neoadjuvant chemoradiation therapy for distal bile duct cancer. Mol Clin Oncol. 2022;16(6):109. 10.3892/mco.2022.2542 PMC9112400 35620208

[42] de Pinho Favaro MT, Atienza-Garriga J, Martínez-Torró C, Parladé E, Vázquez E, Corchero JL, et al. Recombinant vaccines in 2022: A perspective from the cell factory. Microb Cell Fact. 2022;21(1):203. 10.1186/s12934-022-01929-8 PMC9532831 36199085

[43] Englesbe MJ, Patel SP, He K, Lynch RJ, Schaubel DE, Harbaugh C, et al. Sarcopenia and mortality after liver transplantation. J Am Coll Surg. 2010;211(2):271–8. 10.1016/j.jamcollsurg.2010.03.039 PMC2914324 20670867

[44] Montano-Loza AJ, Meza-Junco J, Prado CM, Lieffers JR, Baracos VE, Bain VG, et al. Muscle wasting is associated with mortality in patients with cirrhosis. Clin Gastroenterol Hepatol. 2012;10(2):166–73, 73.e1. 10.1016/j.cgh.2011.08.028 21893129

[45] Tandon P, Ney M, Irwin I, Ma MM, Gramlich L, Bain VG, et al. Severe muscle depletion in patients on the liver transplant wait list: Its prevalence and independent prognostic value. Liver Transpl. 2012;18(10):1209–16. 10.1002/lt.23495 22740290

[46] Wang W, Wang H, Yao T, Li Y, Yi L, Gao Y, et al. The top 100 most cited articles on COVID-19 vaccine: A bibliometric analysis. Clin Exp Med. 2023;23(6):2287–99. 10.1007/s10238-023-01046-9 PMC10026222 36939968

[47] Carey EJ, Lai JC, Wang CW, Dasarathy S, Lobach I, Montano-Loza AJ, et al. A multicenter study to define sarcopenia in patients with end-stage liver disease. Liver Transpl. 2017;23(5):625–33. 10.1002/lt.24750 PMC5762612 28240805

[48] Golse N, Bucur PO, Ciacio O, Pittau G, Sa Cunha A, Adam R, et al. A new definition of sarcopenia in patients with cirrhosis undergoing liver transplantation. Liver Transpl. 2017;23(2):143–54. 10.1002/lt.24671 28061014

[49] Dasarathy S, Merli M. Sarcopenia from mechanism to diagnosis and treatment in liver disease. J Hepatol. 2016;65(6):1232–44. 10.1016/j.jhep.2016.07.040 PMC5116259 27515775

[50] Ponziani FR, Picca A, Marzetti E, Calvani R, Conta G, Del Chierico F, et al. Characterization of the gut-liver-muscle axis in cirrhotic patients with sarcopenia. Liver Int: Off J International Assoc Study Liver. 2021;41(6):1320–34. 10.1111/liv.14876 33713524

[51] Ohsawa Y, Ohtsubo H, Munekane A, Ohkubo K, Murakami T, Fujino M, et al. Circulating α-Klotho counteracts transforming growth factor-β-induced sarcopenia. Am J Pathol. 2023;193(5):591–607. 10.1016/j.ajpath.2023.01.009 36773783

[52] Zhang T, Cheng JK, Hu YM. Gut microbiota as a promising therapeutic target for age-related sarcopenia. Ageing Res Rev. 2022;81:101739. 10.1016/j.arr.2022.101739 36182084

[53] Kamo N, Kaido T, Uozumi R, Ito T, Yagi S, Hata K, et al. Effect of administration of β-hydroxy-β-methyl butyrate-enriched formula after liver transplantation: A pilot randomized controlled trial. Nutrition. 2020;79-80:110871. 10.1016/j.nut.2020.110871 32593895

[54] van Vugt JLA, Alferink LJM, Buettner S, Gaspersz MP, Bot D, Darwish Murad S, et al. A model including sarcopenia surpasses the MELD score in predicting waiting list mortality in cirrhotic liver transplant candidates: A competing risk analysis in a national cohort. J Hepatol. 2018;68(4):707–14. 10.1016/j.jhep.2017.11.030 29221886

[55] Lai Q, Magistri P, Lionetti R, Avolio AW, Lenci I, Giannelli V, et al. Sarco-Model: A score to predict the dropout risk in the perspective of organ allocation in patients awaiting liver transplantation. Liver Int: Off J International Assoc Study Liver. 2021;41(7):1629–40. 10.1111/liv.14889 33793054

[56] Bi WL, Hosny A, Schabath MB, Giger ML, Birkbak NJ, Mehrtash A, et al. Artificial intelligence in cancer imaging: Clinical challenges and applications. CA Cancer J Clin. 2019;69(2):127–57. 10.3322/caac.21552 PMC6403009 30720861

[57] Kim YJ. Machine learning models for sarcopenia identification based on radiomic features of muscles in computed tomography. Int J Env Res Public Health. 2021;18(16):8710. 10.3390/ijerph18168710 PMC8394435 34444459

[58] Lu D, Lin Z, Wang R, Chen Z, Zhuo J, Xu L, et al. Multi-omics profiling reveals Chitinase-3-like protein 1 as a key mediator in the crosstalk between sarcopenia and liver cancer. Redox Biol. 2022;58:102538. 10.1016/j.redox.2022.102538 PMC9682348 36417796

[59] Lu D, Hu Z, Chen H, Khan AA, Xu Q, Lin Z, et al. Myosteatosis and muscle loss impact liver transplant outcomes in male patients with hepatocellular carcinoma. J Cachexia Sarcopenia Muscle. 2024;15(5):2071–83. 10.1002/jcsm.13554 PMC11446693 39192518

[60] Liu Z, Wu Y, Khan AA, Lun LU, Wang J, Chen J, et al. Deep learning-based radiomics allows for a more accurate assessment of sarcopenia as a prognostic factor in hepatocellular carcinoma. J Zhejiang Univ Sci B. 2024;25(1):83–90. 10.1631/jzus.B2300363 PMC10758209 38163668

[61] Oost LJ, Kustermann M, Armani A, Blaauw B, Romanello V. Fibroblast growth factor 21 controls mitophagy and muscle mass. J Cachexia Sarcopenia Muscle. 2019;10(3):630–42. 10.1002/jcsm.12409 PMC6596457 30895728

